# Chemical composition and anti-microbial potential of essential oils from morphologically distinct *Salvia rosmarinus* (Spenn.) cultivars from Kashmir, India

**DOI:** 10.3389/fmicb.2025.1579383

**Published:** 2025-07-02

**Authors:** Nafeesa Farooq Khan, Abid Bashir, Khursheed Ahmad Ganaie, Sumaya Quadir Shah, Romaan Nazir, Phalisteen Sultan, Qazi Parvaiz Hassan

**Affiliations:** Plant Molecular Biology and Biotechnology, CSIR-IIIM (Branch), Srinagar, India

**Keywords:** *Salvia rosmarinus*, secondary metabolites, anti-microbial, phenotypic traits, gene pool

## Abstract

**Introduction:**

The Kashmir Himalaya, renowned for its rich floristic diversity, harbors a multitude of native and introduced aromatic and medicinal plants. Among these, *Salvia rosmarinus* (rosemary), a Mediterranean native plant species, known for its culinary and therapeutic properties, is widely being cultivated owing to its local adaptability. *Salvia rosmarinus* essential oil has been used in folk medicine, pharmaceutical, and cosmetic industries. In our study, we compiled the morphological and chemoprofiling differences of field grown cultivars, wherein populations were grouped into 21 classes. Further, oils from identified accessions were screened for their anti-microbial potential against panel of four priority pathogens.

**Methods and results:**

The characterization was based on phenotypic traits (flower color variability, calyx color, flower size, and leaf morphology) variance across identified genotypes was validated using Chi 2 test. Abundance distribution data displayed polymorphism in evaluated character/traits of rosemary accessions and a total of 21 classes were reported from an underrepresented region. Furthermore, field grown *Salvia rosmarinus* cultivars in Kashmir Himalaya produced essential oil yield ranging from 0.8% to 1.7% maintaining benchmark constituents. Similarly, variability in chemical constituents using Gas Chromatography Mass Spectrometry (GC/MS), grouped accessions into chemotypes rich in beta-myrcene, 1,8 cineole, and camphor. Antimicrobial assays on the essential oils obtained from different accessions using gram-negative and gram-positive bacteria and one fungal pathogen were conducted to directly evaluate the IC_50_ (concentration at which there is 50 percent growth inhibition of pathogen) and Minimum Inhibitory concentration (MIC) values. MIC evaluation of the active essential oil was performed using the broth dilution method.

**Discussion:**

The data generated in this study emphasizes the use of morphological and chemical characteristics to characterize and conserve elite *Salvia rosmarinus* cultivars, promoting cultivar R1 (1.7%) in summer season and R14 (0.95%) and R3 (0.93%) in winter season for large-scale cultivation, emphasizing propagation of higher essential oil yielding varieties in Kashmir Himalaya. The diverse rosemary genepool conserved in Kashmir exhibits significant variability in essential oil yield and composition while, certain accessions demonstrate potent antimicrobial properties. The findings of the study are useful for further elaborate studies on the development of natural bioactive compounds to improve human health.

## 1 Introduction

Globally, *Salvia rosmarinus* is recognized as a medicinal and aromatic plant species widely used as a fresh or dried herb. Since botanical sources of medicines started garnering attention, *Salvia rosmarinus* has been cultivated worldwide for its vital role in treatment, therapy, drug development, culinary and ornamental applications (Miguel et al., 2007; Gowda et al., 2024). Although plants thrive under a wide range of environmental conditions (Kadri et al., 2011), ecologically, this shrub plant species is well suited to flourish in dry climates and is predominantly found in the Mediterranean region, despite which it shows vast differences in its distributional range. In Kashmir Himalayan region this plant is an introduced crop considering the importance of the biochemical constituents present in it. Often, abiotic and biotic factors create distinctness in terms of plant taxonomical traits, such as flower color, leaf shape, leaf size, and overall growth habits (Herrera, 2005; Gowda et al., 2024). Such variability in morphological traits such as flower color was previously reported by Nunziata et al. (2019), but with a resurgence of interest and search for bioactive compounds of the plant in healthcare, a recent report illustrated significant differences in taxonomical features such as flowers and leaves in this plant (Gowda et al., 2024). Consequently, these variations in morphological traits, under the influence of abiotic and biotic factors, are increasingly utilized to identify elite *Salvia rosmarinus* cultivars with superior characteristics. Amongst the known methods of cultivar identification, morphological identification, biochemical identification, identification of signature components in essential oil, nutritional assessment, and molecular marker determination are the most popular techniques to optimize selection of elite cultivars (Sadeh et al., 2019; Gowda et al., 2024).

Quantitative and qualitative screening of essential oils often reveals significant variability and acts as a strong determinant in cultivar screening (Bradley, 2006). Relationships based on the chemical constituents conferring bioactivity to the extract and essential oil remains are also important criteria for cultivar selection (Baratta et al., 1998; Jiang et al., 2011) shaped by environmental factors (Piccaglia et al., 1993). Although specific compound isolation from a particular cultivar, along with its specific activity, remains to be investigated (Santoyo et al., 2005; Ivanovic et al., 2012), *Salvia rosmarinus* is undergoing large-area expansion cultivation. However, assessment remains a critical aspect for identifying elite cultivars, such as breeding enhancement and germplasm conservation (Paterson et al., 1991, Gowda et al., 2024). Further improvements in the sales of herbal products, and the inherent antimicrobial and antifungal activities of *Salvia rosmarinus*, has imposed an additional pressure on the identification of cultivars. Moreover, large-scale propagation in India necessitates the assessment of plant genetic diversity across biogeographical regions, especially visa viz underrepresented region like Kashmir Himalaya. Thus, in modern times the characterization and classification of chemotype-based identification and morphometric analysis have been central to similar studies as efficacy, safety, and quality are of utmost importance (Zigene et al., 2022). Zia et al. (2017) emphasized that such studies are important and effective in germplasm conservation. Screening of cultivars based on chemoprofile, such as high levels of 1,8-cineole (Sadeh et al., 2019) and α-pinene, boranyl acetate, myrcene, or verbenone as signature components of *Salvia rosmarinus*, has been reported previously (Satyal et al., 2017). Despite the above-mentioned important criterion that the plant has different chemotype populations, *Salvia rosmarinus* is not well characterized in terms of its morphology or chemotypic composition in the study region. To address this knowledge gap, the current study aimed to characterize the adapted population of *Salvia rosmarinus* cultivars by analyzing their essential oil chemotypic composition and provide a morphometric description of the plant that could ultimately help identify stable and established *Salvia rosmarinus* cultivars essential for the sustainable use of molecular methods for varietal development.

## 2 Materials and methods

### 2.1 Study area and plant material collection

For the present study, *Salvia rosmarinus* plants were collected from CSIR-IIIM fields in Srinagar in 2023–2024 during summer and autumn seasons, and 21 accessions were tagged and labeled after proper morphometric analysis of the fields based on morphological dissimilarities. The plant material was mounted on an herbarium sheet to obtain accession numbers and for future references. The physical characteristics of the randomly selected 50 individual plants were recorded before they were tagged in the field.

### 2.2 Essential oil screening

Fresh plant material from morphological distinct tagged accessions of *Salvia rosmarinus* weighing 300 g was harvested from CSIR-IIIM field/farms. The sample material (300 g approx. of each accession) was chopped finely and subjected to hydro-distillation using Clevenger apparatus (Perfit, India). All samples were processed for extraction of oil for 6 h in triplicates. The essential oil was collected in vials, and excess water was removed either by a micro-syringe or using anhydrous sodium sulfate (HiMedia). The oil obtained was stored in refrigerator at 4°C till Gas Chromatography/Mass Spectrometry downstream analysis was performed. Comparative percentage yield of oil was calculated as volume of oil extracted to the weight of plant material used for extraction of oil (v/w).

### 2.3 Downstream analysis by GC/MS

The essential oil compounds were identified using GC/MS. A 10 μL sample, prepared with methanol, was subjected to GC/MS to determine the molecular weight and retention time of the compounds. The experiment was performed using an Agilent 7890 A gas chromatograph coupled with an Agilent 5975 C Inert XL MSD mass spectrometer. The data were processed using MassHunter Workstation software (USA) following a method outlined by Manzoor et al. (2021). Helium was used as the carrier gas at a flow rate of 0.5 mL/min. The temperature program was adjusted as 50°C for 1 min, followed by an increase to 250°C at a rate of 50°C/min, maintaining the final temperature for 4 min. The mass spectra were scanned within the range of 50–600 amu, with the ionization energy set at 70 eV and a scan speed of 0.5 s per scan. Full proof of identity of the compounds present in the essential oil was obtained using the Wiley and NIST libraries in conjunction with key compound identification. The peak area percentages were recorded without applying correction factors.

### 2.4 Screening antimicrobial potential of essential oils

The essential oils obtained from each accession through hydro-distillation were evaluated for their antimicrobial potential. The cultivars with high oil yield (quantitative measure) were evaluated for their bioactive potential against few priority pathogens using the microbroth dilution method. Concentration of essential oil used in evaluation was 1 μL/mL, which was serially diluted. Microtiter plate assay was conducted in triplicates. Bacterial (Gram-positive and negative) pathogens (*Staphylococcus aureus*, *Staphylococcus dysenteriae*, and *Bacillus cereus*) and one fungal pathogen (*Candida albicans*) were grown in microtiter plates with different concentrations of the oil in Mueller–Hinton broth for bacterial pathogens and Potato Dextrose Broth (PDB) for fungal pathogens (HiMedia, Mumbai, India) at 37°C for 24 h. IC_50_ and MIC of the oils were calculated (Bashir et al., 2023). Nystatin and ciprofloxacin (HiMedia) were used as positive controls. Oil was used in micrograms per milliliter concentration and antibiotics were used in microliters per milliliter unit.

### 2.5 Data analysis and presentation

Morphometric analysis of selected plant species was worked out and phenotypic traits were recorded for study accessions. Qualitative traits subjected to Chi-Square (X2) test (*p* < 0.05) using XL STAT package in MS Excel to determine association between varieties and characters or whether they are independent. Chi square test was evaluated against tabulated values at significance value of 0.05 and calculating degree of freedom from formula df = (rows-1 × column-1). A dendrogram (tree diagram illustrating hierarchical relationships) was constructed using multivariate analysis based on the neighbor-joining method. The linkage analysis was performed using Bray–Curtis similarity to assess the relationships among the accessions. Chemical compound structures were generated using BIOVIA DRAW 2019. Further, the effect of the essential oil on pathogens are presented as means ± standard deviation (SD), based on three independent biological replicates, each containing the same number of technical replicates.

## 3 Results

### 3.1 Morphological characterization of accessions

**FIGURE 1:**
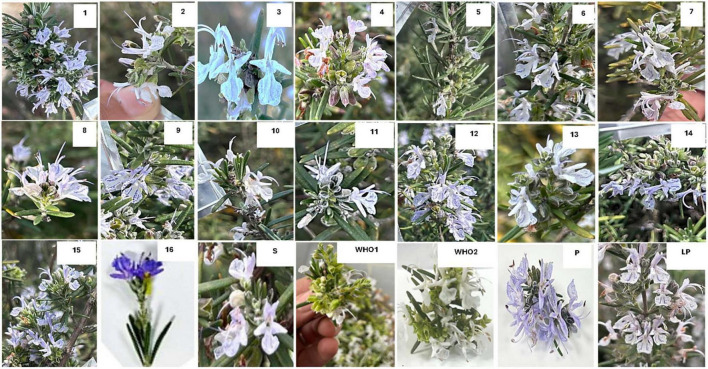
Floral traits of accessions characterized in this study.

Morphologically, the identified varieties were characterized on five traits including flower petal color, flower size, leaf type, leaf color, and sepal color (Figure 1). Amongst accessions flowers, displayed peculiar characteristics, showing variability in size and color. Perusal of data showed variation in flower color, flowering time, and leaf color. Flowers exhibit six phenotypic classes. Amongst these, prevalence of light blue flowers (Accession R2, R3, R5, R6, R7, R9, R10, R15) was predominant, followed by bluish white (R12), white (R11, R13, R14), purple (R16, P), and light purple (R1, R4) (Supplementary Table 1). Flowers with blue and purple pigments yielded low amounts of essential oils ranging from 0.4% to 0.62% in fall and 0.3 to 1.3% in Summer. In comparison to blue and purple flower plants, light purple (Accessions) plants yielded higher percentage of oil. Plants with light leaf color yielded a higher oil percentage than plants with a dark deep green color (Accessions R1, R3, R6, R9, R12, R15, R16 and L Pur). Supplementary Table 1 provides a detailed description of the traits used for weighing and characterizing the *Salvia rosmarinus* plants along with ascribed accession names.

### 3.2 Comprehensive screening of essential oil from accessions

**FIGURE 2:**
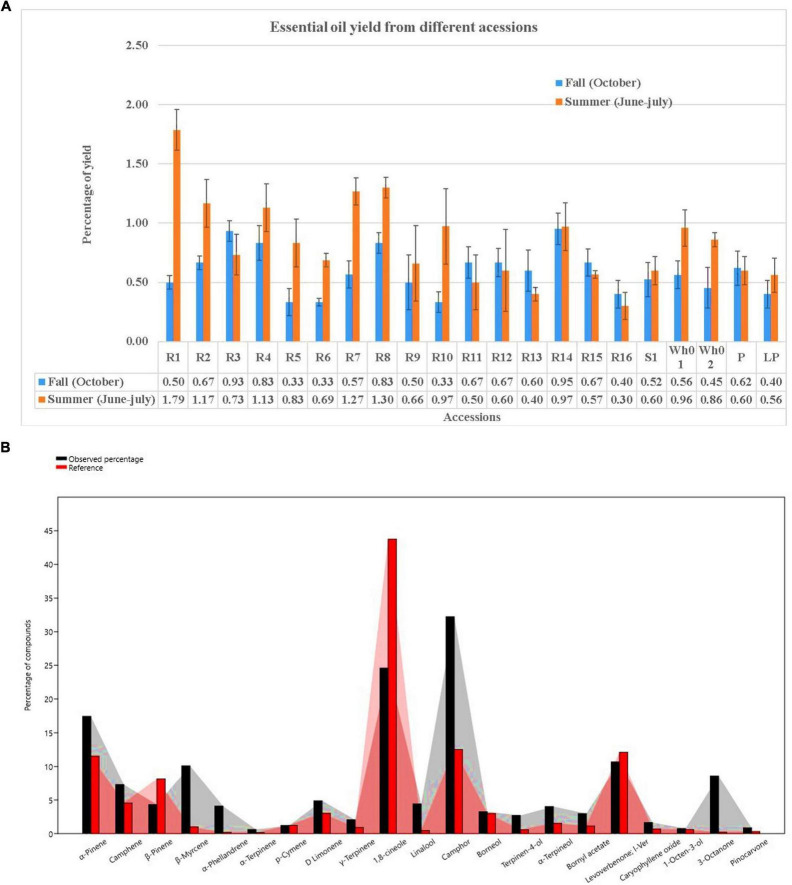
**(A)** Seasonal comparison of essential oil yield in accessions during Fall and Summer. **(B)** Thorough chemoprofiling based on reference compounds.

**FIGURE 3:**
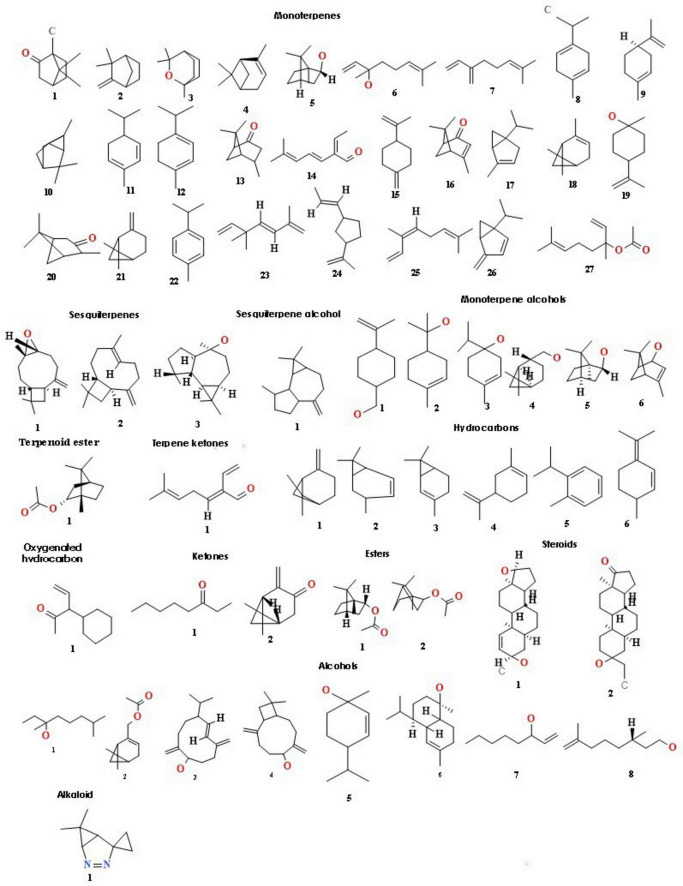
Structures of key volatile compounds present in the essential oil of Rosemary, including: monoterpenes (1–27), sesquiterpenes (1–3), a sesquiterpene alcohol (1), monoterpene alcohols (6), terpenoid esters (1–2), a terpene ketone (1), hydrocarbons (6), an oxygenated hydrocarbon (1), ketones (1–2), esters (1–2), steroids (1–2), alcohols (8), and an alkaloid (1).

**FIGURE 4:**
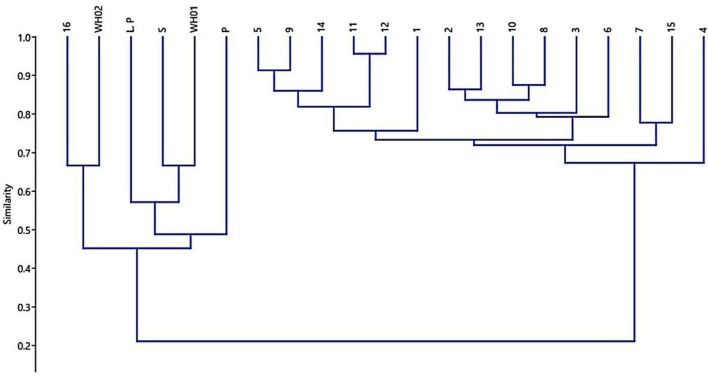
Dendrogram generated using Bray-Curtis similarity to depict the chemoprofile-based relationships among 21 accessions based on their essential oil composition 14.

Essential oil yields varied among the cultivars identified and ranged from 0.4% to 1.7% (v/w). Figure 2A illustrates the yields per cultivar during the summer and autumn seasons, while Figure 2B provides a comparative percentage composition of major compounds present in the essential oil across *Salvia rosmarinus* essential oil accessions. The comparison among different essential oil accessions revealed varying percentages of oil yield, with a marked decline in essential oil content during the winter season. The highest yield was obtained from accession R14 (0.95%), and the lowest from R5 and R6 (0.33% each) in autumn, whereas in peak summer season, R1 yielded the highest amount of essential oil (1.7%) and the lowest yield was reported from R16 (0.3%). The essential oil screened by GC/MS showed 61 compounds abundantly present in the essential oil, and their descriptions and structures are presented in Figure 3 and Table 1. The major components of the oil are monoterpenes, monoterpene alcohols, sesquiterpenes, sesquiterpene alcohols, terpenoid esters, terpene ketones, hydrocarbons, oxygenated hydrocarbons, ketones, esters, alcohols, steroids, and alkaloids (Table 1). The chemical components identified in the essential oils (Table 2) varied across cultivars. Furthermore, Bray–Curtis similarity-based cluster analysis revealed the formation of two major clusters, grouping the accessions according to the compound composition of their essential oils (Figure 4). A comparison of the identified compounds with those reported in previous studies (Chalchat et al., 1993; Aziz et al., 2022) revealed that several accessions contained higher levels of key reference compounds in their essential oils, which includes highest levels of camphor (32.24 area percentage), α-pinene (17.45), β-myrcene (10.1), levoverbenone, l-verbenone, and D-verbenone (10.68) (Figure 4 and Supplementary Table 2).

**TABLE 1:** Characterization of major identified compounds across accession at > 80% similarity with retention time and area percentage using GC/MS.

S. No	Name	Pub Chem ID	MF^b^	M.W^c^	RT^d^	Area (%)	Similarity^e^
**A**	**Terpenes**						
**I Monoterpenes**							
1	(+)-2-bornanone	159055	C_10_H_16_O	152.23 g/mol	6.185	26.74	96
2	Camphene	129848108	C_10_H_16_	136.23 g/mol	4.033	5.64	96
3	Eucalyptol	2758	C_10_H_18_O	154.25 g/mol	4.916	11.84	94
4	(1R)-2,6,6-Trimethylbicyclo[3.1.1]hept-2-ene	6654	C_10_H_16_	136.23 g/mol	3.866	8.41	96
5	Bicyclo[2.2.1]heptan-2-ol, 1,7,7- trimethyl-, (1S-endo)	439569	C_10_H_18_O	154.25 g/mol	3.866	8.41	96
6	Linalool	6549	C_10_H_18_O	154.25 g/mol	5.596	2.24	97
7	Beta-myrcene	131715234	C_10_H_16_	31.32 g/mol	4.41	2.78	96
8	Gamma-terpinene	7461	C_10_H_16_	136.23 g/mol	5.173	1.71	96
9	D-limonene	440917	C_10_H_16_	136.23 g/mol	4.862	4.24	93
10	Tricyclo[2.2.1.0(2,6)]heptane, 1,3,3-trimethyl	79022	C_10_H_16_	136.23 g/mol	3.753	0.46	95
11	Alpha-phellandrene	7460	C_10_H_16_	136.23 g/mol	0.26	0.26	95
12	1,3-Cyclohexadiene, 1-methyl-4-(1-methylethyl)	7462	C_10_H_16_	136.23 g/mol	4.725	0.61	95
13	Bicyclo[3.1.1]heptan-3-one, 2,6,6- trimethyl-, (1.alpha.,2.beta.,5.alpha.)	84532	C_10_H_16_O	152.23 g/mol	6.458	2.76	84
14	3,5-Heptadienal, 2-ethylidene-6-methyl	572127	C_10_H_14_O	150.22 g/mol	8.492	0.08	89
15	Cyclohexane, 1-methylene-4-(1-methylethenyl)	68140	C_10_H_16_	136.23 g/mol	4.878	9.19	88
16	Bicyclo[3.1.1]hept-3-en-2-one, 4,6,6- trimethyl-, (1S)	29025	C_10_H_14_O	150.22 g/mol	6.819	6.75	96
17	Bicyclo[3.1.0]hex-2-ene, 2-methyl-5-(1-methylethyl)	17868	C_10_H_16_	136.23 g/mol	4.597	1.53	94
18	Alpha-pinene	6654	C_10_H_16_	136.23 g/mol	3.863	5.38	96
19	P-Menth-8-en-1-ol, stereoisomer	8748	C_10_H_18_O	154.25 g/mol	5.665	0.24	94
20	Bicyclo[3.1.1]heptan-3one, 2,6,6-trimethyl	11038	C_10_H_16_O	152.23 g/mol	6.458	2.76	84
21	Beta. pinene	14896	C_10_H_16_	136.23 g/mol	9.9	2.75	89
22	P-cymene	7463	C_10_H_14_	134.22 g/mol	1.2	1.2	95
23	1,3,6-Heptatriene, 2,5,5-trimethyl	5320377	C_10_H_16_	136.23 g/mol	7.318	0.05	95
24	1-Isopropenyl-3-propenyl cyclopentane	5369943	C_11_H_18_	150.26 g/mol	5.9	0.27	84
25	1,3,6-Octatriene,3,7- dimethyl-, (Z)	5320250	C_10_H_16_	136.23 g/mol	5.022	0.22	97
26	Bicyclo[3.1.0]hex-2-ene, 4-methylene-1-(1-methylethyl)	524198	C_10_H_14_	134.22 g/mol	0.22	0.22	94
27	Linalyl acetate	8294	C_12_H_20_O_2_	196.29 g/mol	7.124	96	96
**II Sesquiterpene**							
1	Caryophyllene oxide	1742210	C_15_H_24_O	220.35 g/mol	10.218	0.54	96
2	Caryophyllene	5281515	C_15_H_24_	204.35 g/mol	8.811	1.01	96
3	(−)-Globulol	12304985	C_15_H_26_O	222.37 g/mol	10.77	0.74	83
**III Sesquiterpene alcohol**							
1	1H-Cycloprop[e]azulene, decahydro-1,1,7-trimethyl-4-methylene	91354	C_15_H_24_	224.35 g/mol	5.9	0.27	86
**IV Monoterpene alcohol**							
1	Alpha-terpineol	17100	C_10_H_18_O	154.25 g/mol	6.592	3.05	96
2	Terpinen-4-ol	11230	C_10_H_18_O	154.25 g/mol	6.456	1.97	93
3	(−)-cis-myrtanol	11084102	C_10_H_18_O	154.25 g/mol	7.063	0.39	86
4	Isoborneol	6552009	C_10_H_18_O	154.25 g/mol	15.701	7.29	91
5	Verbenol	92874	C_10_H_14_O	150.22 g/mol	6.095	0.39	90
6	Shisool	519954	C_10_H_18_O	154.25 g/mol	7.126	0.18	86
**V Terpenoid ester**							
1	Bicyclo[2.2.1]heptan-2-ol,1,7,7- trimethyl-, acetate, (1S-endo)	93009	C_12_H_20_O_2_	196.29 g/mol	19.083	3.85	84
**VI Terpene ketone**							
1	Cis-ocimene, 8-oxo-	5369951	C_10_H_14_O	150.22 g/mol	17.012	5.26	82
**B**	**Hydrocarbon**						
1	Bicyclo[3.1.1]heptane, 6,6-dimethyl-2- methylene-, (1S)	14896	C_10_H_16_	136.23 g/mol	4.326	2.7	95
2	(+)-4-carene	530422	C_10_H_16_	136.23 g/mol	4.728	0.44	95
3	2-carene	79044	C_10_H_16_	136.23 g/mol	5.495	0.84	96
4	Cyclohexene, 1-methyl-5-(1-methylethenyl)-, (R)	102625	C_10_H_16_	136.23 g/mol	7.88	7.88	89
5	O-Cymene	10703	C_10_H_14_	134.22 g/mol	4.812	1.44	95
6	Cyclohexene, 3-methyl-6-(1-methylethylidene)	102443	C_10_H_16_	136.23 g/mol	5.495	1.27	96
**C**	**Oxygenated hydrocarbon**						
1	4-Penten-2-one, 3-cyclohexyl	41542	C_11_H_18_O	166.26 g/mol	11.579	41.25	84
**D**	**Ketone**						
1	3-octanone	246728	C_8_H_16_O	128.21 g/mol	4.359	0.57	90
2	Pinocarvone	1231431	C_10_H_14_O	150.22 g/mol	6.318	0.52	87
**E**	**Ester**						
1	Bornyl acetate	93009	C_12_H_20_O_2_	196.29 g/mol	7.5	0.93	96
2	Acetic acid, 1,7,7-trimethyl-bicyclo[2.2.1]hept-2-yl ester	6448	C_12_H_20_O_2_	196.29 g/mol	7.499	0.26	95
**F**	**Alcohol**						
1	3-octanol	6548	C_10_H_22_O	158.28 g/mol	4.464	0.64	95
2	Bicyclo[3.1.1]hept-2-ene-2-methanol, 6,6-dimethyl	61262	C_12_H_18_O_2_	194.27 g/mol	6.669	0.45	95
3	(1R,7S,E)-7-Isopropyl-4,10-dimethylenecyclodec-5-enol	13304974	C_15_H_24_O	220.35 g/mol	10.77	0.17	83
4	Caryophylla-4(12),8(13)-die5.alpha-ol	527418	C_15_H_24_O	220.35 g/mol	10.627	0.1	85
5	4-Isopropyl-1-methylcyclohex-2-enol	526657	C_10_H_18_O	154.25 g/mol	5.62	0.2	92
6	Alpha-cadinol	6431302	C_15_H_26_O	222.37 g/mol	10.737	0.07	90
7	1-Octen-3-ol	18827	C_8_H_16_O	128.21 g/mol	4.292	0.74	92
8	7-Octen-1-ol, 3,7- dimethyl-, (S)	81263	C_10_H_20_O	156.26 g/mol	6.872	0.38	95
**G**	**Steroid**						
1	5.alpha.-androst-1-ene-3.beta.,17.beta.-diol	11300765	C_19_H_30_O_2_	290.4 g/mol	10.771	0.7	83
2	Androstan-17-one, 3-ethyl-3- hydroxy-, (5.alpha.)	14681481	C_21_H_34_O_2_	318.5 g/mol	10.767	0.16	83
**H**	**Alkaloid**						
1	Spiro (6,6-dimethyl-2,3-diazobicyclo [3.1.0] hex-2-ene-4,1′-cyclopropane)	534124	C_8_H_12_N_2_	136.19 g/mol	9.899	2.33	83

A–H Nature of identified compounds.

*^b^*Molecular formula.

*^c^*Molecular weight.

*^d^*Retention time.

*^e^*Similarity greater than 80% and area percentage > 0.5%.

**TABLE 2:** Compounds and their occurrence in all the samples (*compounds with area percentage > 0.5).

Compounds*	1	2	3	4	5	6	7	8	9	10	11	12	13	14	15	16	LP	*P*	S	WH01	WH02
(+)-2-Bornanone	1	1	1	1	1	1	1	1	1	1	1	1	1	1	1	1	1	1	1	1	1
(1R)-2,6,6-Trimethylbicyclo[3.1.1]hept-2-ene	1	1	1	1	1	1	1	1	1	1	1	1	1	1	0	0	1	1	1	1	0
(+)-4-carene	0	1	0	0	1	0	0	1	1	0	1	1	1	1	0	0	0	0	0	0	0
alpha.-terpineol	1	1	1	1	1	1	1	1	1	1	1	1	1	1	1	0	0	0	0	0	0
(1R,7S,E)-7-Isopropyl-4,10-dimethylenecyclodec-5-enol	0	0	0	0	1	0	0	1	0	1	0	0	0	1	0	0	0	0	0	0	0
(−)-Globulol	0	0	0	0	0	0	1	0	0	0	0	0	0	0	0	0	0	0	0	0	0
Beta-myrcene	1	1	1	1	1	1	1	1	1	1	1	1	1	1	1	0	0	0	0	0	0
Gamma-terpinene	1	1	1	1	1	1	1	1	1	1	1	1	0	1	1	0	0	0	0	0	0
Eucalyptol	1	1	1	1	1	1	1	1	1	1	1	1	1	1	1	1	1	1	0	1	1
1,3-Cyclohexadiene, 1-methyl-4-(1-methylethyl)-	0	0	1	0	0	0	0	0	0	0	0	0	0	0	1	0	0	0	0	0	0
Camphene	1	1	1	1	1	1	1	1	1	1	1	1	1	1	1	1	1	1	1	1	1
Bicyclo[2.2.1]heptan-2-ol, 1,7,7- trimethyl-, (1S-endo)-	1	1	1	1	1	1	1	1	1	1	1	1	1	1	1	0	0	1	0	0	0
Alpha-phellandrene	0	0	0	0	1	0	0	1	1	0	1	1	0	0	0	1	0	0	0	0	0
Linalool	1	1	1	1	1	1	1	1	1	1	1	1	1	1	1	0	0	0	0	0	0
3-Octanone	0	1	1	1	0	1	0	1	0	1	0	0	1	1	1	0	0	0	0	0	0
3-Octanol	0	1	1	0	0	1	0	1	0	1	0	0	1	0	0	0	0	0	0	0	0
Bicyclo[3.1.1]hept-3-en-2-one, 4,6,6- trimethyl-, (1S)-	1	1	1	1	1	1	1	1	1	1	1	1	1	1	1	0	0	0	0	0	0
Bicyclo[3.1.1]heptane, 6,6-dimethyl-2- methylene-, (1S)-	1	1	1	1	1	0	1	1	1	1	1	1	1	1	1	0	0	0	0	0	0
Bornyl acetate	1	1	0	1	1	0	0	1	0	0	1	1	1	1	1	0	0	0	0	0	0
Terpinen-4-ol	1	1	1	0	1	1	1	1	1	1	1	1	1	1	1	0	0	0	0	0	0
D-Limonene	1	1	0	1	0	1	1	1	0	1	0	0	1	0	1	0	0	0	0	0	0
o-Cymene	0	1	1	0	1	1	1	1	1	1	1	1	1	1	1	0	0	0	0	0	0
Caryophyllene	1	0	0	0	1	0	1	1	1	1	1	1	0	1	1	1	0	0	0	0	1
Caryophyllene oxide	1	0	0	1	1	1	1	1	1	1	1	1	0	1	1	0	0	0	0	0	0
Alpha-cadinol	0	0	0	0	1	0	0	0	1	0	0	0	0	1	1	0	0	0	0	0	0
Pinocarvone	0	1	1	1	1	1	1	1	1	1	0	0	1	1	1	0	0	0	0	0	0
Cyclohexene, 3-methyl-6-(1-methylethylidene)-	1	1	1	0	1	0	1	0	1	1	1	1	0	1	1	0	0	0	0	0	0
2-Carene	0	0	0	1	0	1	0	1	0	0	0	0	1	0	0	0	0	0	0	0	0
p-Cymene	1	0	0	0	0	0	0	0	0	0	0	0	0	0	0	0	0	0	0	0	0
Shisool	0	0	0	1	0	0	0	0	0	0	0	0	0	0	1	0	0	0	0	0	0
Linalyl acetate	0	0	0	0	0	0	0	0	0	0	0	0	1	0	0	0	0	0	0	0	0
7-Octen-1-ol, 3,7- dimethyl-, (S)-	0	0	0	1	0	0	1	0	0	0	1	1	0	0	0	0	0	0	0	0	0
4-Isopropyl-1-methylcyclohex-2-enol	0	0	0	0	0	0	0	0	0	0	1	1	0	0	0	0	0	0	0	0	0
Bicyclo[3.1.1]hept-2-ene-2-methanol, 6,6-dimethyl-	0	1	0	1	0	1	1	0	0	1	0	0	0	1	0	0	0	0	0	0	0
Bicyclo[3.1.0]hex-2-ene, 2-methyl-5-(1-methylethyl)-	0	0	1	0	0	1	1	0	0	0	0	0	0	1	1	0	0	0	0	0	0
1-Octen-3-ol	0	0	0	0	0	0	1	0	0	0	0	0	0	0	1	0	0	0	0	0	0
Caryophylla-4(12),8(13)-dien-5.alpha.-ol	0	0	0	0	0	0	1	0	0	0	0	0	0	0	1	0	0	0	0	0	0
1,3,6-Octatriene, 3,7- dimethyl-, (Z)-	0	0	0	1	0	0	0	0	0	0	0	0	0	0	0	0	0	0	0	0	0
Tricyclo[2.2.1.0(2,6)]heptane, 1,3,3-trimethyl-	0	1	0		0	0	0	0	0	0	0	0	0	0	0	0	0	0	0	0	0
(−)-cis-myrtanol	0	0	0	0	0	0	1	0	0	0	0	0	0	0	0	0	0	0	0	0	0
Bicyclo[3.1.1]heptan-3-one, 2,6,6- trimethyl-, (1.alpha.,2.beta.,5.alpha.)-	0	0	0	1	0	0	0	0	0	0	0	0	0	0	0	0	0	0	0	0	0
5.alpha.-Androst-1-ene-3.beta.,17.beta.-diol	0	0	0	1	0	0	0	0	0	0	0	0	0	0	0	0	0	0	0	0	0
1-Isopropenyl-3-propenyl cyclopentane	0	0	0	0	0	1	0	0	0	0	0	0	0	0	0	0	0	0	0	0	0
Cyclohexane, 1-methylene-4-(1-methylethenyl)-	0	0	0	0	0	0	0	0	0	0	0	1	0	0	0	0	0	0	0	0	0
Cyclohexene, 1-methyl-5-(1-methylethenyl)-, (R)-	0	0	0	0	0	0	0	0	0	0	1	0	0	0	0	0	0	0	0	0	0
Bicyclo[3.1.0]hex-2-ene, 4-methylene-1-(1-methylethyl)-	0	0	0	0	0	0	0	0	1	0	0	0	0	0	0	0	0	0	0	0	0
1H-Cycloprop[e]azulene, decahydro-1,1,7-trimethyl-4-methylene-	0	0	0	0	0	0	0	0	0	0	0	0	0	1	0	0	0	0	0	0	0
Androstan-17-one, 3-ethyl-3- hydroxy-, (5.alpha.)-	0	0	0	0	0	0	0	0	0	0	0	0	0	0	1	0	0	0	0	0	0
1,3,6,10-Dodecatetraene, 3,7,11- trimethyl-, (Z,E)-	0	0	0	0	0	0	0	0	0	0	0	0	0	0	0	0	1	0	0	0	0
Propanoic acid, 3- hydroxy-, hydrazide	0	0	0	0	0	0	0	0	0	0	0	0	0	0	0	0	1	0	0	0	0
p-Menth-8-en-1-ol, stereoisomer	0	0	0	0	0	0	0	0	0	0	0	0	0	0	0	0	0	0	0	0	0
1,3,6-Heptatriene, 2,5,5-trimethyl-	0	0	0	0	0	0	0	0	0	0	0	0	0	0	0	0	0	0	0	0	0
2,3-Dimethyl-cyclohexa-1,3-diene	0	0	0	0	0	0	0	0	0	0	0	0	0	0	0	0	0	0	0	0	0
2,6-Dimethyl-1,3,6-heptatriene	0	0	0	0	0	0	0	0	0	0	0	0	0	0	0	0	0	0	0	0	0
Verbenol	0	0	0	0	0	0	0	0	1	0	0	0	0	1	0	0	0	0	0	0	1
Bicyclo[3.1.1]heptan-3-one, 2,6,6-trimethyl-	0	0	0	1	0	0	0	0	0	0	0	0	0	0	0	0	0	0	0	0	0
4-Penten-2-one, 3-cyclohexyl-	0	0	0	0	0	0	0	0	0	0	0	0	0	0	0	0	0	0	1	0	0
Cyclohexanol, 2-methyl-5-(1-methylethenyl)-	0	0	0	0	0	0	0	0	0	0	0	0	0	0	0	0	0	0	0	0	0
Bicyclo[3.1.1]hept-3-en-2-one, 4,6,6-trimethyl-	0	0	0	0	0	0	0	0	0	0	0	0	0	0	0	0	0	0	0	0	0
10-Undecyn-1-ol	0	0	0	0	0	0	0	0	0	0	0	0	0	0	0	0	0	0	0	0	0
cis-ocimene, 8-oxo-	0	0	0	0	0	0	0	0	0	0	0	0	0	0	0	0	0	0	1	0	0
Bicyclo[7.2.0]undec-4-ene, 4,11,11-trimethyl-8- methylene-,[1R-(1R*,4Z,9S*)]-	0	0	0	0	0	0	0	0	0	0	0	0	0	0	0	0	0	0	0	0	0
Acetic acid, 1,7,7-trimethyl-bicyclo[2.2.1]hept-2-yl ester	0	0	0	0	0	1	0	0	0	0	0	0	0	0	0	0	0	0	1	1	0
Acetic acid, hydrazide	0	0	0	0	0	0	0	0	0	0	0	0	0	0	0	0	0	1	0	0	0
Alpha-pinene	0	0	0	0	0	0	0	0	0	0	0	0	0	0	1	1	0	0	0	0	1
Hydrazinecarboxamide	0	0	0	0	0	0	0	0	0	0	0	0	0	0	0	0	0	0	0	1	0
Beta-pinene	0	0	0	0	0	0	0	0	0	0	0	0	0	0	0	1	0	1	0	0	0
1,3,7-Octatriene, 3,7-dimethyl-	0	0	0	0	0	0	0	0	0	0	0	0	0	0	0	1	0	0	0	0	0
Isoborneol	0	0	0	0	0	0	0	0	0	0	0	0	0	0	0	1	1	0	1	1	1
3,5-Heptadienal, 2-ethylidene-6-methyl-	0	0	0	0	0	0	0	0	0	0	0	0	1	0	0	1	0	0	0	0	0
Spiro (6,6-dimethyl-2,3-diazobicyclo [3.1.0] hex-2-ene-4,1′-cyclopropane)	0	0	0	0	0	0	0	0	0	0	0	0	0	0	0	0	1	0	1	0	1
Benzene, tetradecyl-	0	0	0	0	0	0	0	0	0	0	0	0	0	0	0	0	1	0	0	0	0
Bicyclo[2.2.1]heptan-2-ol, 1,7,7- trimethyl-, acetate, (1S-endo)-	0	0	0	0	0	0	0	0	0	0	0	0	0	0	0	0	1	1	0	0	0
Total compounds	53	52	48	43	39	45	52	46	48	46	41	45	47	55	51	10	10	8	8	8	8

*> 0.5 percent.

A variable number of compounds were obtained from the accessions (Figure 3). Higher number of volatiles were reported from accessions R1, R2, R3, R7, R9, R10, R12, R13, R14, and R15. Using GC/MS analyses, 138 volatiles were identified as constituents of the investigated essential oils across the 21 accessions (Supplementary Table 1). In-depth analysis revealed the chemoprofiles of cineole-camphor, verbenone, linalool, and borneol chemotypes. The 1,8-cineole and camphor content were higher in summer and winter for all accessions. Overall, accessions growing in this region exhibited higher concentrations of monoterpenes i.e., 27 compounds out 138 compounds were monoterpenes (Table 1). In autumn, the essential oil constituents displayed the presence of 33 major compounds as compared to the average of 100 compounds in summer.

### 3.3 Antimicrobial effect of volatiles

The effect of the essential oils on pathogen inhibition resulted in variable degrees of activity. This variation, detected through IC_50_ and MIC values, exhibited differences in the quality of oils from different source accessions. Oil extract from different accessions inhibited three of the four pathogens tested [including *S. aureus*, *S. dysenteriae*, *B. cereus* (bacterial pathogens), and *C. albicans* (a fungal pathogen)] with IC_50_ of < 50 μl/ml. While most oils displayed substantial inhibition of *S. dysenteriae*, *B. cereus*, and *C. albicans* growth, all sample oils showed minimal inhibitory effects against *S. aureus*. *C. albicans* showed potential inhibition by oil extract from accessions R1, R2, R3, R4 R9, R10, R14, R13, and R16 with an IC_50_ ranging from 6.5 to 48.6 μl/ml (Table 3). The oil samples from Accessions R3, R2, R11, R12, R13, R14, R15, R16, and WHO1 were effective against Gram-positive bacterium *B. cereus* with IC_50_ ranging from 6.9 to 36.2 μl/ml (Table 3). However, the Gram-positive bacterium *S. aureus* was only slightly inhibited by oil accessions R4, R5, and R6, whereas Gram-negative bacterium *S. dysenteriae* was inhibited by oil samples with IC_50_ ranging from 9.5 to 48.5 μL ml^–1^. Furthermore, the antifungal activity against *C. albicans* reported MIC of 12.2 μl/mL. Similarly, the antibacterial activity against Gram-positive (*B. cereus*) and Gram-negative pathogen (*S. dysenteriae*) showed susceptibility to the MIC of *Salvia rosmarinus* essential oil at 8 and 15 μl/mL concentrations, respectively. Our findings show that oils from accessions R4, R5, and R6 possess antimicrobial properties that are effective in controlling pathogen development against all four test pathogens, albeit with erratic IC_50_ values. However, R1, R2, R3, R8, R12, R14, R15, and R16 and the most effective R4 were found to have inhibitory effects on gram-negative bacteria. Moreover, R1, R2, R3, R4, R6, R11, R12, R13, R14, R15, R16, and WH01 exhibited maximum inhibition of gram-positive bacteria.

**TABLE 3:** Pathogenic inhibition effect of essential oil on selected pathogens.

Oils from chemotypes	*S. aureus* (Gram + ve) ATCC 29213		*S. dysenteriae* (Gram -ve) NCTC 11,311		*B. cereus* (Gram + ve) IIIM 25		*C. albicans MTCC 474	
	IC_50_	MIC	IC_50_	MIC	IC_50_	MIC	IC_50_	MIC
	**μL/mL**							
LP Bornyl acetate/camphor/1,8-cineole/α-pinene/camphene type	> 100	> 100	64.0 ± 10.4	> 100	82.6 ± 5.1	> 100	48.6 ± 3.2	> 100
P 1,8-cineole/α-pinene/camphor type	> 100	> 100	69.9 ± 3.8	> 100	71.2 ± 3.1	> 100	34.2 ± 6.8	75.6
S 4-Penten-2-one, 3-cyclohexyl-/camphor type	> 100	> 100	> 100	> 100	71.2 ± 3.1	> 100	75.2 ± 5.2	> 100
R1 β-Myrcene/camphor/1,8-cineole type	> 100	> 100	21.4 ± 2.5	45	35 ± 4.4	72.3	9.2 ± 2.1	19.5
R2 1,8-cineole/α-pinene/camphor type	> 100	> 100	11.6 ± 0.7	19.4	7.1 ± 0.35	13.5	6.5 ± 0.48	12.2
R3 1,8-cineole/camphor type	> 100	> 100	13.6 ± 2.7	27.5	6.9 ± 0.5	12.8	7.2 ± 0.5	13.8
R4 1,8-cineole/camphor type	78.0 ± 5.2	> 100	9.5 ± 0.8	15	8.1 ± 1.0	17.5	8.3 ± 0.7	15.7
R5 1,8-cineole/α-pinene/camphor	66.2 ± 5.0	> 100	29.4 ± 3.4	62.1	66.3 ± 4.3	> 100	47.8 ± 2.2	> 100
R6 1,8-cineole/camphor type	54.6 ± 2.8	> 100	86.0 ± 3.7	> 100	35.4 ± 2.0	73.8	50.3 ± 2.5	> 100
R7 1,8-cineole/camphor/verbenone type	> 100	> 100	54.4 ± 2.4	> 100	68.6 ± 8.4	> 100	45.2 ± 4.4	> 100
R8 1,8-cineole/camphor/type	> 100	> 100	21.5 ± 2.3	39.5	68.9 ± 8.5	> 100	43.7 ± 1.7	> 100
R9 1,8-cineole/α-pinene/camphor type	> 100	> 100	48.5 ± 4.7	> 100	78.8 ± 5	> 100	21.0 ± 1.6	44.5
R10 1,8-cineole/α-pinene type	> 100	> 100	46.6 ± 6.0	> 100	47.8 ± 2.2	> 100	17.2 ± 0.97	35
R11 1,8-cineole/camphor type	> 100	> 100	58.1 ± 1.0	> 100	28.7 ± 3.0	65.2	38.7 ± 6.9	78
R12 camphor type	> 100	> 100	12.1 ± 1.2	25.2	7.5 ± 1.1	15.8	10.8 ± 1.6	19.9
R13 1,8-cineole/camphor type	> 100	> 100	29.6 ± 5.0	60.5	21.1 ± 5.6	43.8	28.8 ± 4.6	61.2
R14 1,8-cineole/camphor type	> 100	> 100	11.0 ± 0.5	21.2	10.0 ± 1.0	20.5	11.3 ± 0.86	22.3
R15 1,8-cineole/camphor type	> 100	> 100	11.1 ± 0.6	21.8	8.2 ± 1.2	15.8	32.9 ± 1.3	64.4
R16 1,8-cineole/α-pinene/camphor type	> 100	> 100	12.3 ± 4.1	25.8	8.4 ± 0.5	17.1	10.5 ± 0.6	20.5
WHO1 1,8-cineole/camphor type	> 100	> 100	24.5 ± 0.37	52.1	36.2 ± 6.0	75.5	35.9 ± 1.0	75
WHO2 1,8-cineole/α-pinene/camphor type	> 100	> 100	54.7 ± 10.1	> 100	56.6 ± 3.1	> 100	66.7 ± 8.2	> 100
Ciprofloxacin/*nystatin	0.362 ± 0.09	0.57	0.12 ± 0.009	0.21	0.002 ± 0.0001	0.05	1.5 ± 0.022	2.6

Ciprofloxacin/*nystatin were used in micrograms per milliliter. *Means antibiotics used in experiment.

## 4 Discussion

The Kashmir Himalayan region is a non-native region for aromatic herbs such as *Salvia rosmarinus*. The region is now showing increased cultivation practices for this aromatic plant on a larger scale. A reference database for identifying elite genotypes based on 21 accessions growing in the Kashmir Himalayan region during summer and autumn was documented in this study. A total of 50 individuals randomly selected from different accessions were screened from densely growing populations. This study classified entire accessions of rosemary (*Salvia rosmarinus*) based on a comprehensive evaluation of morphometric traits, including flower color and size, leaf size, color, and type, as well as sepal pigmentation (presence or absence of anthocyanins). These traits hold practical significance for breeding programs aimed at selecting desired phenotypes. A substantial degree of morphological divergence was observed among the accessions. Notably, quantitative traits were predominantly used to estimate genetic variability within the gene pool (Bonny et al., 2019; Zigene et al., 2022), as these traits are primarily influenced by allelic differences and less so by environmental pressures (Briggs and Knowles, 1967; Van Hintum et al., 2000; Zigene et al., 2022). Morphological trait analyses have been pivotal in identifying genetic diversity, thereby aiding the selection and enhancement of cultivars. Among the observed growth habits, upright growth was most prevalent, followed by prostrate and semi-erect forms, aligning with earlier findings by Carrubba et al. (2020) and Zigene et al. (2022), who also documented these three growth types in *S. rosmarinus*. Prostrate forms are often cultivated for higher leaf biomass, whereas upright forms, which yield less foliage and essential oil, are generally favored for ornamental use. Consequently, leaf characteristics such as size and color significantly influence breeding targets aimed at enhancing essential oil yield.

Lattoo et al. (2008) advocated for the use of integrated morphological, phytochemical, and molecular markers to identify elite germplasm. Their findings demonstrated a wide spectrum of flower colors—blue, white, and purple—whose variation was attributable to genetic factors, developmental stages, pigment composition, and environmental variables, including soil pH and UV exposure. For example, in *Xanthoceras sorbifolium*, petal bases shift from yellow to purple during maturation due to anthocyanin accumulation (Lu et al., 2022). Similarly, in *Antirrhinum majus*, petal color varies with pigment composition chlorophylls, carotenoids, and flavonoids which are modulated through different developmental phases (Nabipour Sanjbod et al., 2023). UV light also plays a role, as differing exposure levels can enhance photopigment accumulation, affecting visual cues for pollinators sensitive to UV wavelengths (Valenta et al., 2020). In addition, the study documented leaf size variations among different *S. rosmarinus* cultivars, with lengths ranging from 2 to 4 cm and widths between 2 and 5 mm (World Health Organization [WHO], 1999). Despite these size differences, no major divergence was observed in other morphological features such as leaf texture (leathery), shape (linear to lanceolate), and presence of a distinct aroma (World Health Organization [WHO], 1999). Such size variation may reflect the interplay of genetic regulation and environmental influence (Tisné et al., 2013). Light-responsive pigment regulation contributes to leaf color, aiding photosynthesis, and stress protection (Biswal et al., 2011). Furthermore, environmental shifts, such as soil composition or UV exposure, can induce adaptive changes in leaf morphology and pigmentation (Singh et al., 2006; Goslee et al., 2009; Zhao et al., 2016). The study also assessed seasonal variability in essential oil yield across accessions maintained at CSIR-IIIM, Srinagar, using a standardized extraction protocol under controlled experimental conditions. Significant variation in yield was recorded. Accession R1 exhibited the highest yield (1.7%) in the summer, while accessions R14 (0.95%) and R3 (0.93%) showed relatively higher yields in winter. These results are consistent with the findings of Salido et al. (2003), who reported yields ranging from 1% to 1.8%, with peak production occurring during summer. The elevated yields in warmer months may be attributed to enhanced volatile compound synthesis driven by environmental variables such as temperature and humidity. The seasonal variation in essential oil content was also accompanied by differences in chemical composition, supporting the notion that oil yield is strongly influenced by both chemotype and morphological traits, including leaf and flower color (da Silva et al., 2003; Sajjadi and Ghannadi, 2012). Lower yields were noted during full flowering and toward the end of the growing season, a pattern similarly noted by Salido et al. (2003), though contrary to earlier reports by Moretti et al. (1998). This study revealed notable differences in the chemical composition of essential oils from various *S. rosmarinus* accessions, influenced both by seasonal changes and inherent genetic variability. Major constituents included camphor (32.24%), 1,8-cineole (24.59%), α-pinene (17.45%), verbenone (10.68%), β-myrcene (10.1%), 3-octanone (8.58%), linalool (4.4%), and borneol (3.25%). This chemotypic profile aligns with previous studies identifying camphor as a dominant compound (Daferera et al., 2000). These profiles directly influence the oil’s therapeutic and aromatic properties, enhancing its utility in traditional medicine and aromatherapy. Although morphological traits showing significant differences between varieties but alone are insufficient to identify elite cultivars, a significant correlation was found between chemoprofile and flower/leaf color. Cluster analysis based on Bray–Curtis similarity grouped accessions into two distinct clusters, indicating underlying genetic variation even among accessions sourced from the same region. Several minor yet significant compounds—3-octanone, endo-borneol, alpha-phellandrene, 3-octanol, fenchol, pinocarvone, caryophyllene oxide, and bornyl acetate—were present in higher concentrations than reported in earlier literature (Supplementary Table 2), suggesting potential chemical enrichment. Among the 21 major constituents commonly identified in rosemary essential oil, the following were notable: alpha-ocimene, cyclofenchene, isopinocamphone, 1-androstenediol, cyclopentane derivatives, spathulenol, bergamiol, and globulol, among others (Dellacassa et al., 1999; Daferera et al., 2000; Celiktas et al., 2007; Laborda et al., 2013; Jordán et al., 2013). These findings support the classification of *S. rosmarinus* essential oils into cineoliferum, camphoriferum, and verbenoniferum chemotypes (Napoli et al., 2010). Notably, the composition of the present samples closely resembled Moroccan rosemary oil, which is rich in 1,8-cineole (43.5%–57.7%), differing from French and Spanish variants typically high in α-pinene (19.4%–35.1%) (Chalchat et al., 2011). The essential oils from different accessions exhibited potent antimicrobial activity, assessed through MIC and IC_50_ values against four pathogenic bacteria. Oils from most accessions inhibited three out of four test organisms effectively, primarily due to their bioactive constituents. Consistent with findings by Kabotso et al. (2024), the principal antimicrobial components included eucalyptol, camphor, and endo-borneol. MIC and MBC values ranged from 3.13 to 6.25 mg/mL and 3.12 to 12.5 mg/mL, respectively, indicating promising antibacterial potential. Accession R1, characterized by a β-myrcene/camphor/1,8-cineole profile, showed inhibitory effects at concentrations of 21.4, 35, and 9.2 μL/mL against specific pathogens. Similarly, accessions R2 and R3 (1,8-cineole/α-pinene/camphor type) demonstrated robust antimicrobial properties, as did R12 (camphor type). These observations are in line with earlier reports attributing such activity to phenolic and flavonoid compounds (Meccatti et al., 2021). The antibacterial effects of rosemary oil against *S. aureus*, *B. cereus*, *E. coli*, and *Pseudomonas aeruginosa* have also been documented (Jafarı-Sales and Pashazadeh, 2020; Abdul Rahman, 2015). In addition, the tested essential oils were effective against *Shigella dysenteriae*, further supporting their broad-spectrum antibacterial properties. While preliminary results suggest high antimicrobial efficacy, further studies are necessary to evaluate therapeutic applications. The present study focused on MIC and IC_50_ evaluations as preliminary indicators of antimicrobial activity. In future studies, the therapeutic potential will be further assessed through comprehensive analyses, including time-kill kinetics, synergy assays, cytotoxicity profiling, and benchmarking against clinical reference strains.

## 5 Conclusion

This study documented the morphological and chemical variability of *Salvia rosmarinus* cultivated in the Kashmir Himalayas, with morphological characterization serving as an first and foremost classification approach which can be combined with chemical profiles of essential oil to describe the varieties growing in the region. Field-grown *Salvia rosmarinus* (syn. *Rosmarinus officinalis*) in Kashmir’s temperate Himalaya produced essential oil yields (0.88%–1.70%) that exceeded the 0.8%–1.5% range reported for its Mediterranean native region, while maintaining the benchmark camphor/1,8-cineole chemotype. Oil dominantly showed presence of Monoterpenes, Sesquiterpene, and Monoterpene alcohol. The study identified few potential cultivars with better essential oil yield and chemoprofiles. In nutshell, field grown cultivars also yielded satisfactory amount of essential oil under unfavorable winter conditions. A subset of Kashmir accessions shows markedly stronger antibacterial activity (MIC = 4% v/v against *Staphylococcus aureus*) than many commercial reference oils, positioning these high-altitude cultivars as a globally competitive, climate-resilient source of pharmaceutical and flavor grade rosemary oil. This result confirms potential use of essential oil against priority pathogens. Evidently, essential oil rich in β-myrcene, camphor, and 1,8-cineole, exhibited the strongest antimicrobial activity against three test pathogens. While 1,8-cineole/α-pinene/camphor type and a camphor-type, also displayed antimicrobial effects against these pathogens. Moreover, tested oil samples exerted minimal effect inhibitory activity against on *Staphylococcus aureus*. Our findings from this study highlight the potential of *Salvia rosmarinus* essential oils cultivars, as natural antimicrobial agents. Considering that the identified accessions have a significant inhibitory role in pathogenesis, these accessions require the identification of active compounds for further investigation into their mechanisms of action against other priority pathogens.

## Data Availability

The original contributions presented in this study are included in this article/Supplementary material, further inquiries can be directed to the corresponding authors.
