# Supplementary material for: Chemical composition and anti-microbial potential of essential oils from morphologically distinct Salvia rosmarinus (Spenn.) cultivars from Kashmir, India
Source: Front Microbiol. 2025 Jul 2;16:1579383. doi: 10.3389/fmicb.2025.1579383 (PMC12263608; doi:10.3389/fmicb.2025.1579383)
Supplement: Supplementary file 1 [file Table_1.docx]

**Supplementary table 1:** Abundance distribution and chi square test of qualitative traits from rosmeary accessions.

|  | **Character / Traits** | **Accessions** | | | | | | | | | | | | | | | | | | | | | |
| --- | --- | --- | --- | --- | --- | --- | --- | --- | --- | --- | --- | --- | --- | --- | --- | --- | --- | --- | --- | --- | --- | --- | --- |
|  | **Observed values** | **R1** | **R2** | **R3** | **R4** | **R5** | **R6** | **R7** | **R8** | **R9** | **R10** | **R11** | **R12** | **R13** | **R14** | **R15** | **R16** | **LIGHT PURPLE** | **PURPLE** | **SMALL** | **WH01** | **WH02** | **Total** |
| **Flower Petal Colour** | **Light Purple (LP)** | 11 | 0 | 6 | 4 | 0 | 0 | 0 | 0 | 0 | 0 | 0 | 0 | 0 | 0 | 0 | 0 | 0 | 0 | 0 | 0 | 0 | **21** |
|  | **Light Blue (LB)** | 0 | 1 | 0 | 0 | 1 | 2 | 1 | 0 | 1 | 1 | 0 | 0 | 0 | 0 | 4 | 0 | 0 | 0 | 0 | 0 | 0 | **11** |
|  | **Blue (B)** | 0 | 0 | 0 | 0 | 0 | 0 | 0 | 2 | 0 | 0 | 0 | 0 | 0 | 0 | 0 | 1 | 0 | 1 | 0 | 0 | 0 | **4** |
|  | **Blue White (BW)** | 0 | 0 | 0 | 0 | 0 | 0 | 0 | 0 | 0 | 0 | 0 | 2 | 0 | 0 | 0 | 0 | 0 | 0 | 0 | 0 | 2 | **4** |
|  | **Purple (P)** | 0 | 0 | 0 | 0 | 0 | 0 | 0 | 0 | 0 | 0 | 0 | 0 | 0 | 0 | 0 | 0 | 1 | 0 | 0 | 0 | 0 | **1** |
|  | **White (W)** | 0 | 0 | 0 | 0 | 0 | 0 | 0 | 0 | 0 | 0 | 1 | 0 | 2 | 2 | 0 | 0 | 0 | 0 | 2 | 2 | 0 | **9** |
|  | **total** | **11** | **1** | **6** | **4** | **1** | **2** | **1** | **2** | **1** | **1** | **1** | **2** | **2** | **2** | **4** | **1** | **1** | **1** | **2** | **1** | **1** | **50** |
|  | **Expected Value** | **R1** | **R2** | **R3** | **R4** | **R5** | **R6** | **R7** | **R8** | **R9** | **R10** | **R11** | **R12** | **R13** | **R14** | **R15** | **R16** | **LIGHT PURPLE** | **PURPLE** | **SMALL** | **WH01** | **WH02** |  |
|  | **Light Purple (LP)** | 4.62 | 0.42 | 2.52 | 1.68 | 0.42 | 0.84 | 0.42 | 0.84 | 0.42 | 0.42 | 0.42 | 0.84 | 0.84 | 0.84 | 1.68 | 0.42 | 0.42 | 0.42 | 0.84 | 0.42 | 0.42 |  |
|  | **Light Blue (LB)** | 2.42 | 0.22 | 1.32 | 0.88 | 0.22 | 0.44 | 0.22 | 0.44 | 0.22 | 0.22 | 0.22 | 0.44 | 0.44 | 0.44 | 0.88 | 0.22 | 0.22 | 0.22 | 0.44 | 0.22 | 0.22 |  |
|  | **Blue (B)** | 0.88 | 0.08 | 0.48 | 0.32 | 0.08 | 0.16 | 0.08 | 0.16 | 0.08 | 0.08 | 0.08 | 0.16 | 0.16 | 0.16 | 0.32 | 0.08 | 0.08 | 0.08 | 0.16 | 0.08 | 0.08 |  |
|  | **Blue white (BW)** | 0.88 | 0.08 | 0.48 | 0.32 | 0.08 | 0.16 | 0.08 | 0.16 | 0.08 | 0.08 | 0.08 | 0.16 | 0.16 | 0.16 | 0.32 | 0.08 | 0.08 | 0.08 | 0.16 | 0.08 | 0.08 |  |
|  | **Purple (P)** | 0.22 | 0.02 | 0.12 | 0.08 | 0.02 | 0.04 | 0.02 | 0.04 | 0.02 | 0.02 | 0.02 | 0.04 | 0.04 | 0.04 | 0.08 | 0.02 | 0.02 | 0.02 | 0.047 | 0.02 | 0.02 |  |
|  | **White (W)** | 1.98 | 0.18 | 1.08 | 0.72 | 0.18 | 0.36 | 0.18 | 0.36 | 0.18 | 0.18 | 0.18 | 0.36 | 0.36 | 0.36 | 0.72 | 0.18 | 0.18 | 0.18 | 0.23 | 0.18 | 0.18 |  |
| **p value = 2.36E-20** | | | | | | | | | | | | | | | | | | | | | | | |
| **Flower size** | **OBSERVED** | R1 | **R2** | **R3** | **R4** | **R5** | **R6** | **R7** | **R8** | **R9** | **R10** | **R11** | **R12** | **R13** | **R14** | **R15** | **R16** | **LIGHT PURPLE** | **PURPLE** | **SMALL** | **WH01** | **WH02** | **Total** |
|  | **Very Small (VS)** | 6 | 0 | 0 | 0 | 0 | 0 | 0 | 1 | 0 | 0 | 0 | 0 | 0 | 0 | 0 | 0 | 0 | 0 | 0 | 0 | 4 | **11** |
|  | **Small (S)** | 0 | 4 | 5 | 1 | 0 | 0 | 1 | 0 | 1 | 1 | 1 | 0 | 1 | 0 | 5 | 0 | 0 | 0 | 1 | 0 | 0 | **21** |
|  | **large (L)** | 0 | 0 | 0 | 0 | 2 | 2 | 0 | 0 | 0 | 0 | 0 | 1 | 0 | 0 | 0 | 0 | 0 | 0 | 0 | 5 | 0 | **10** |
|  | **Intermediate size (IS)** | 0 | 0 | 0 | 0 | 0 | 0 | 0 | 0 | 0 | 0 | 0 | 0 | 0 | 5 | 0 | 1 | 1 | 1 | 0 | 0 | 0 | **8** |
|  | **total** | **6** | **4** | **5** | **1** | **2** | **2** | **1** | **1** | **1** | **1** | **1** | **1** | **1** | **5** | **5** | **1** | **1** | **1** | **1** | **5** | **4** | **50** |
|  | **EXPECTED** |  |  |  |  |  |  |  |  |  |  |  |  |  |  |  |  |  |  |  |  |  |  |
|  | **flower size** | **R1** | **R2** | **R3** | **R4** | **R5** | **R6** | **R7** | **R8** | **R9** | **R10** | **R11** | **R12** | **R13** | **R14** | **R15** | **R16** | **LIGHT PURPLE** | **PURPLE** | **SMALL** | **WH01** | **WH02** |  |
|  | **Very Small (VS)** | 1.32 | 0.88 | 1.1 | 0.22 | 0.44 | 0.44 | 0.22 | 0.22 | 0.22 | 0.22 | 0.22 | 0.22 | 0.22 | 1.1 | 1.1 | 0.22 | 0.22 | 0.22 | 0.22 | 1.1 | 0.88 |  |
|  | **Small (S)** | 2.52 | 1.68 | 2.1 | 0.42 | 0.84 | 0.84 | 0.42 | 0.42 | 0.42 | 0.42 | 0.42 | 0.42 | 0.42 | 2.1 | 2.1 | 0.42 | 0.42 | 0.42 | 0.42 | 2.1 | 1.68 |  |
|  | **large (L)** | 1.2 | 0.8 | 1 | 0.2 | 0.4 | 0.4 | 0.2 | 0.2 | 0.2 | 0.2 | 0.2 | 0.2 | 0.2 | 1 | 1 | 0.2 | 0.2 | 0.2 | 0.2 | 1 | 0.8 |  |
|  | **Intermediate size (IS)** | 0.96 | 0.64 | 0.8 | 0.16 | 0.32 | 0.32 | 0.16 | 0.16 | 0.16 | 0.16 | 0.16 | 0.16 | 0.16 | 0.8 | 0.8 | 0.16 | 0.16 | 0.16 | 0.16 | 0.8 | 0.64 |  |
|  | **p value= 1.1612E-09** | | | | | | | | | | | | | | | | | | | | | | |
| **Sepal colour** | **OBSERVED** | **R1** | **R2** | **R3** | **R4** | **R5** | **R6** | **R7** | **R8** | **R9** | **R10** | **R11** | **R12** | **R13** | **R14** | **R15** | **R16** | **LIGHT PURPLE** | **PURPLE** | **SMALL** | **WH01** | **WH02** | **Total** |
|  | **Small and thin (stn)** | 1 | 1 | 0 | 0 | 0 | 0 | 0 | 0 | 0 | 0 | 0 | 0 | 0 | 0 | 0 | 5 | 0 | 0 | 0 | 0 | 0 | **7** |
|  | **Long (lon)** | 0 | 0 | 1 | 0 | 0 | 0 | 0 | 0 | 0 | 0 | 0 | 0 | 0 | 0 | 0 | 0 | 0 | 0 | 0 | 0 | 0 | **1** |
|  | **Small and thick (stk)** | 0 | 0 | 0 | 5 | 0 | 0 | 0 | 0 | 0 | 0 | 0 | 0 | 0 | 1 | 0 | 0 | 0 | 0 | 5 | 0 | 0 | **11** |
|  | **Intermediate/thick (itk)** | 0 | 0 | 0 | 0 | 5 | 0 | 0 | 0 | 1 | 0 | 0 | 0 | 0 | 0 | 5 | 0 | 0 | 0 | 0 | 0 | 0 | **11** |
|  | **Long/thin (ltn)** | 0 | 0 | 0 | 0 | 0 | 1 | 0 | 0 | 0 | 0 | 1 | 1 | 1 | 0 | 0 | 0 | 5 | 0 | 0 | 0 | 0 | **9** |
|  | **Large/very thin (lvtn)** | 0 | 0 | 0 | 0 | 0 | 0 | **1** | **5** | **0** | **0** | **0** | **0** | **0** | **0** | **0** | **0** | **0** | **0** | **0** | **0** | **0** | **6** |
|  | **Large/Thick (ltk)** | 0 | 0 | 0 | 0 | 0 | 0 | 0 | 0 | 0 | 1 | 0 | 0 | 0 | 0 | 0 | 0 | 0 | 0 | 0 | 1 | 0 | **2** |
|  | **Large, thick, and broad (ltkb)** | 0 | 0 | 0 | 0 | 0 | 0 | 0 | 0 | 0 | 0 | 0 | 0 | 0 | 0 | 0 | 0 | 0 | 1 | 0 | 0 | 2 | **3** |
|  |  | **1** | **1** | **1** | **5** | **5** | **1** | **1** | **5** | **1** | **1** | **1** | **1** | **1** | **1** | **5** | **5** | **5** | **1** | **5** | **1** | **2** | **50** |
|  | **EXPECTED** | **R1** | **R2** | **R3** | **R4** | **R5** | **R6** | **R7** | **R8** | **R9** | **R10** | **R11** | **R12** | **R13** | **R14** | **R15** | **R16** | **LIGHT PURPLE** | **PURPLE** | **SMALL** | **WH01** | **WH02** |  |
|  | **Small and thin (stn)** | 0.14 | 0.14 | 0.14 | 0.7 | 0.7 | 0.14 | 0.14 | 0.7 | 0.14 | 0.14 | 0.14 | 0.14 | 0.14 | 0.14 | 0.7 | 0.7 | 0.7 | 0.14 | 0.7 | 0.14 | 0.28 |  |
|  | **Long (lon)** | 0.02 | 0.02 | 0.02 | 0.1 | 0.1 | 2.14 | 2.14 | 0.1 | 2.14 | 2.14 | 2.14 | 2.14 | 2.14 | 2.14 | 0.1 | 0.1 | 0.1 | 2.14 | 10.7 | 2.14 | 4.28 |  |
|  | **Small and thick (stk)** | 0.22 | 0.22 | 0.22 | 1.1 | 1.1 | 0.22 | 0.22 | 1.1 | 0.22 | 0.22 | 0.22 | 0.22 | 0.22 | 0.22 | 1.1 | 1.1 | 1.1 | 0.22 | 1.1 | 0.22 | 0.44 |  |
|  | **Intermediate/thick (itk)** | 0.22 | 0.22 | 0.22 | 1.1 | 1.1 | 0.22 | 0.22 | 1.1 | 0.22 | 0.22 | 0.22 | 0.22 | 0.22 | 0.22 | 1.1 | 1.1 | 1.1 | 0.22 | 1.1 | 0.22 | 0.44 |  |
|  | **Long/thin (ltn)** | 0.18 | 0.18 | 0.18 | 0.9 | 0.9 | 0.18 | 0.18 | 0.9 | 0.18 | 0.18 | 0.18 | 0.18 | 0.18 | 0.18 | 0.9 | 0.9 | 0.9 | 0.18 | 0.9 | 0.18 | 0.36 |  |
|  | **Large/very thin (lvtn)** | 0.12 | 0.12 | 0.12 | 0.6 | 0.6 | 0.12 | 0.12 | 0.6 | 0.12 | 0.12 | 0.12 | 0.12 | 0.12 | 0.12 | 0.6 | 0.6 | 0.6 | 0.12 | 0.6 | 0.12 | 0.24 |  |
|  | **Large/Thick (ltk)** | 0.04 | 0.04 | 0.04 | 0.2 | 0.2 | 0.04 | 0.04 | 0.2 | 0.04 | 0.04 | 0.04 | 0.04 | 0.04 | 0.04 | 0.2 | 0.2 | 0.2 | 0.04 | 0.2 | 0.04 | 0.08 |  |
|  | **Large, thick, and broad (ltkb)** | 0.06 | 0.06 | 0.06 | 0.3 | 0.3 | 0.06 | 0.06 | 0.3 | 0.06 | 0.06 | 0.06 | 0.06 | 0.06 | 0.06 | 0.3 | 0.3 | 0.3 | 0.06 | 0.3 | 0.06 | 0.12 |  |
|  | **p value = 6.854E-25** | | | | | | | | | | | | | | | | | | | | | | |
| 4 | **OBSERVED** | **R1** | **R2** | **R3** | **R4** | **R5** | **R6** | **R7** | **R8** | **R9** | **R10** | **R11** | **R12** | **R13** | **R14** | **R15** | **R16** | **LIGHT PURPLE** | **PURPLE** | **SMALL** | **WH01** | **WH02** | **Total** |
| **Leaf colour** | **Light green LG** | 5 | 0 | 5 | 0 | 0 | 1 | 0 | 0 | 1 | 0 | 0 | 1 | 0 | 0 | 1 | 1 | 0 | 0 | 0 | 0 | 5 | **20** |
|  | **Dark green DG** | 0 | 5 | 0 | 0 | 0 | 0 | 1 | 0 | 0 | 1 | 1 | 0 | 1 | 1 | 0 | 0 | 1 | 1 | 5 | 5 | 0 | **22** |
|  | **Green G** | 0 | 0 | 0 | 1 | 1 | 0 | 0 | 6 | 0 | 0 | 0 | 0 | 0 | 0 | 0 | 0 | 0 | 0 | 0 | 0 | 0 | **8** |
|  |  | **5** | **5** | **5** | **1** | **1** | **1** | **1** | **6** | **1** | **1** | **1** | **1** | **1** | **1** | **1** | **1** | **1** | **1** | **5** | **5** | **5** | **50** |
|  | **EXPECTED** | R1 | **R2** | **R3** | **R4** | **R5** | **R6** | **R7** | **R8** | **R9** | **R10** | **R11** | **R12** | **R13** | **R14** | **R15** | **R16** | **LIGHT PURPLE** | **PURPLE** | **SMALL** | **WH01** | **WH02** |  |
|  | **Light green (LG)** | 2 | 2 | 2 | 0.4 | 0.4 | 0.4 | 0.4 | 2.4 | 0.4 | 0.4 | 0.4 | 0.4 | 0.4 | 0.4 | 0.4 | 0.4 | 0.4 | 0.4 | 2 | 2 | 2 |  |
|  | **Dark green (DG)** | 2.2 | 2.2 | 2.2 | 0.44 | 0.44 | 0.44 | 0.44 | 2.64 | 0.44 | 0.44 | 0.44 | 0.44 | 0.44 | 0.44 | 0.44 | 0.44 | 0.44 | 0.44 | 2.2 | 2.2 | 2.2 |  |
|  | **Green (G)** | 0.8 | 0.8 | 0.8 | 0.16 | 0.16 | 0.16 | 0.16 | 0.96 | 0.16 | 0.16 | 0.16 | 0.16 | 0.16 | 0.16 | 0.16 | 0.16 | 0.16 | 0.16 | 0.8 | 0.8 | 0.8 |  |
|  | **p value = 4.7914E-07** | | | | | | | | | | | | | | | | | | | | | | |
| 5 | **OBSERVED** | **R1** | **R2** | **R3** | **R4** | **R5** | **R6** | **R7** | **R8** | **R9** | **R10** | **R11** | **R12** | **R13** | **R14** | **R15** | **R16** | **LIGHT PURPLE** | **PURPLE** | **SMALL** | **WH01** | **WH02** | TOTAL |
| **Sepal colour** | **Green (G)** | 2 | 0 | 0 | 0 | 0 | 0 | 0 | 0 | 0 | 0 | 0 | 0 | 0 | 0 | 6 | 0 | 0 | 0 | 0 | 0 | 0 | **8** |
|  | **Blue (B)** | 0 | 2 | 0 | 0 | 0 | 0 | 0 | 3 | 0 | 0 | 0 | 0 | 0 | 0 | 0 | 0 | 0 | 0 | 0 | 0 | 0 | **5** |
|  | **Bluish green (BG)** | 0 | 0 | 10 | 1 | 1 | 1 | 1 | 0 | 1 | 1 | 1 | 1 | 1 | 1 | 0 | 0 | 0 | 0 | 1 | 1 | 3 | **25** |
|  | **Light green (LG)** | 0 | 0 | 0 | 0 | 0 | 0 | 0 | 0 | 0 | 0 | 0 | 0 | 0 | 0 | 0 | 4 | 5 | 3 | 0 | 0 | 0 | **12** |
|  |  | **2** | **2** | **10** | **1** | **1** | **1** | **1** | **3** | **1** | **1** | **1** | **1** | **1** | **1** | **6** | **4** | **5** | **3** | **1** | **1** | **3** | **50** |
|  | **EXPECTED** | **R1** | **R2** | **R3** | **R4** | **R5** | **R6** | **R7** | **R8** | **R9** | **R10** | **R11** | **R12** | **R13** | **R14** | **R15** | **R16** | **LIGHT PURPLE** | **PURPLE** | **SMALL** | **WH01** | **WH02** |  |
|  | **Green (G)** | 0.32 | 0.32 | 1.60 | 0.16 | 0.16 | 0.16 | 0.16 | 0.48 | 0.16 | 0.16 | 0.16 | 0.16 | 0.16 | 0.16 | 0.96 | 0.64 | 0.80 | 0.48 | 0.16 | 0.16 | 0.48 |  |
|  | **Blue (B)** | 0.20 | 0.20 | 1.00 | 0.10 | 0.10 | 0.10 | 0.10 | 0.30 | 0.1 | 0.1 | 0.1 | 0.1 | 0.1 | 0.1 | 0.60 | 0.40 | 0.50 | 0.30 | 0.10 | 0.10 | 0.30 |  |
|  | **Bluish green (BG)** | 1.00 | 1.00 | 5.00 | 0.50 | 0.50 | 0.50 | 0.50 | 1.50 | 0.5 | 0.5 | 0.5 | 0.5 | 0.5 | 0.5 | 3.00 | 2.00 | 2.50 | 1.50 | 0.50 | 0.50 | 1.50 |  |
|  | **Light green (LG)** | 0.48 | 0.48 | 2.40 | 0.24 | 0.24 | 0.24 | 0.24 | 0.36 | 0.24 | 0.24 | 0.24 | 0.24 | 0.24 | 0.24 | 1.44 | 0.96 | 1.20 | 0.36 | 0.24 | 0.24 | 0.36 |  |
|  | **p value = 3.1001E-11** | | | | | | | | | | | | | | | | | | | | | | |
| **Growth form** | **OBSERVED** | **R1** | **R2** | **R3** | **R4** | **R5** | **R6** | **R7** | **R8** | **R9** | **R10** | **R11** | **R12** | **R13** | **R14** | **R15** | **R16** | **LIGHT PURPLE** | **PURPLE** | **SMALL** | **WH01** | **WH02** | TOTAL |
|  | **Semi errect (SE)** | 2 | 0 | 0 | 0 | 0 | 0 | 0 | 0 | 0 | 0 | 2 | 3 | 3 | 0 | 0 | 0 | 0 | 0 | 0 | 0 | 0 | **10** |
|  | **Upright(UR)** | 0 | 1 | 1 | 1 | 1 | 1 | 1 | 0 | 0 | 3 | 0 | 0 | 0 | 2 | 4 | 2 | 0 | 0 | 0 | 4 | 0 | **21** |
|  | **prostrate (PG)** | 0 | 0 | 0 | 0 | 0 | 0 | 0 | 3 | 4 | 0 | 0 | 0 | 0 | 0 | 0 | 0 | 3 | 3 | 3 | 0 | 3 | **19** |
|  |  | **2** | **1** | **1** | **1** | **1** | **1** | **1** | **3** | **4** | **3** | **2** | **3** | **3** | **2** | **4** | **2** | **3** | **3** | **3** | **4** | **3** | **50** |
|  | **EXPECTED** | **R1** | **R2** | **R3** | **R4** | **R5** | **R6** | **R7** | **R8** | **R9** | **R10** | **R11** | **R12** | **R13** | **R14** | **R15** | **R16** | **LIGHT PURPLE** | **PURPLE** | **SMALL** | **WH01** | **WH02** |  |
|  | **Semi errect (SE)** | 0.40 | 0.20 | 0.20 | 0.20 | 0.20 | 0.20 | 0.20 | 0.6 | 0.8 | 0.6 | 0.4 | 0.6 | 0.6 | 0.4 | 0.8 | 0.4 | 0.6 | 0.6 | 0.6 | 0.8 | 0.6 |  |
|  | **Upright(UR)** | 0.84 | 0.42 | 0.42 | 0.42 | 0.42 | 0.42 | 0.42 | 1.26 | 1.68 | 1.26 | 0.84 | 1.26 | 1.26 | 0.84 | 1.68 | 0.84 | 1.26 | 1.26 | 1.26 | 1.68 | 1.26 |  |
|  | **prostrate (PG)** | 0.76 | 0.38 | 0.38 | 0.38 | 0.38 | 0.38 | 0.38 | 1.14 | 1.52 | 1.14 | 0.76 | 1.14 | 1.14 | 0.76 | 1.52 | 0.76 | 1.14 | 1.14 | 1.14 | 1.52 | 1.14 |  |
| **p value = 4.7914E-07** | | | | | | | | | | | | | | | | | | | | | | | |
| 7 | **OBSERVED** | **R1** | **R2** | **R3** | **R4** | **R5** | **R6** | **R7** | **R8** | **R9** | **R10** | **R11** | **R12** | **R13** | **R14** | **R15** | **R16** | **LIGHT PURPLE** | **PURPLE** | **SMALL** | **WH01** | **WH02** |  |
| **Leaf Length** | **UPTO 2** | 1 | 1 | 0 | 2 | 0 | 0 | 0 | 0 | 0 | 0 | 0 | 0 | 0 | 0 | 0 | 3 | 4 | 0 | 0 | 0 | 0 | **11** |
|  | **2-3cm** | 0 | 0 | 2 | 0 | 2 | 0 | 2 | 2 | 2 | 2 | 2 | 4 | 2 | 0 | 2 | 0 | 0 | 2 | 1 | 5 | 2 | **30** |
|  | **ABOVE 3cm** | 0 | 0 | 0 | 0 | 0 | 2 | 0 | 0 | 0 | 0 | 0 | 0 | 0 | 5 | 0 | 0 | 0 | 0 | 0 | 0 | 0 | **7** |
|  |  | **1** | **1** | **2** | **2** | **2** | **2** | **2** | **2** | **2** | **2** | **2** | **4** | **2** | **5** | **2** | **3** | **4** | **2** | **1** | **5** | **2** | **50** |
|  | **EXPECTED** | **R1** | **R2** | **R3** | **R4** | **R5** | **R6** | **R7** | **R8** | **R9** | **R10** | **R11** | **R12** | **R13** | **R14** | **R15** | **R16** | **LIGHT PURPLE** | **PURPLE** | **SMALL** | **WH01** | **WH02** |  |
|  | **UPTO 2** | 0.22 | 0.22 | 0.25 | 0.25 | 0.25 | 0.25 | 0.25 | 0.25 | 0.25 | 0.25 | 0.44 | 0.88 | 0.5 | 1.10 | 0.5 | 0.66 | 1 | 0.25 | 0.25 | 1.10 | 0.25 |  |
|  | **2-3cm** | 0.60 | 0.60 | 0.58 | 0.58 | 0.58 | 0.58 | 0.58 | 0.58 | 0.58 | 0.58 | 1.20 | 2.40 | 1.15 | 3.00 | 1.15 | 1.80 | 2.3 | 0.58 | 0.58 | 3.00 | 0.58 |  |
|  | **ABOVE 3** | 0.14 | 0.14 | 0.15 | 0.15 | 0.15 | 0.15 | 0.15 | 0.15 | 0.15 | 0.15 | 0.28 | 0.56 | 0.3 | 0.70 | 0.3 | 0.42 | 0.6 | 0.15 | 0.15 | 0.70 | 0.15 |  |
| **P = 8.8765E-13** | | | | | | | | | | | | | | | | | | | | | | | |
|  | | | | | | | | | | | | | | | | | | | | | | | |

**Supplementary Table 2: Major volatiles obtained in higher percentage from all accessions**

| **Essential compounds (major signature compound)** | **Cited reference** | **Reference range** | **Observed**  **(Summer percentage)** | **Observed**  **(Fall percentage)** |
| --- | --- | --- | --- | --- |
| α-Pinene | Rašković et al. 2023 | 11.51 | 13.84 | 16.98 |
| Camphene | Rašković et al. 2023 | 4.55 | 7.27 | 9.42 |
| β-Pinene | Rašković et al. 2023 | 8.16 | 4.31 | 8.3 |
| β-Myrcene | Rašković et al. 2023 | 0.99 | 10.1 | - |
| α-Phellandrene | Rašković et al. 2023 | 0.19 | 4.08 | 1.1 |
| α-Terpinene | Rašković et al. 2023 | 0.14 | 0.61 | - |
| p-Cymene | Rašković et al. 2023 | 1.23 | 1.2 | - |
| D Limonene | [Mwithiga](https://www.cell.com/heliyon/fulltext/S2405-8440(22)00565-5) et al. 2022 | 3.016 | 4.88 | - |
| γ-Terpinene | Rašković et al. 2023 | 0.92 | 2.05 | - |
| 1,8-cineole | Rašković et al. 2023 | 43.77 | 24.59 | 43.42 |
| Linalool | Rašković et al. 2023 | 0.46 | 4.4 | 3.98 |
| Camphor | Rašković et al. 2023 | 12.53 | 32.24 | 41.37 |
| Borneol | Rašković et al. 2023 | 2.97 | 3.25 | - |
| Terpinen-4-ol | Rašković et al. 2023 | 0.56 | 2.69 | - |
| α-Terpineol | Rašković et al. 2023 | 1.53 | 4.02 | - |
| Bornyl acetate | Rašković et al. 2023 | 1.13 | 11.5 | 11.5 |
| Levoverbenone; l-Verbenone; D-Verbenone | Anh et al. 2019 | 12.12 | 10.68 | 1.92 |
| Caryophyllene oxide | [Christopoulou](https://pubmed.ncbi.nlm.nih.gov/?term=%22Christopoulou%20SD%22%5BAuthor%5D) et al. 2021 | 0.66 | 1.64 | - |
| Caryophyllene | Anh et al. 2019 | 2.66 |  | 2.42 |
| 1-Octen-3-ol | Satyal et al. 2017 | 0.6 | 0.74 | - |
| Pinocarvone | Satyal et al. 2017 | 0.3 | 0.85 | - |
| 3-Octanone | Satyal et al. 2017 | 0.2 | 8.58 | - |
| 4-Penten-2-one, 3-cyclohexyl- | - | Not defined | Not Defined | 41.25 |
| β-Terpineol | Nowak et al. 2013 | 0.02 | - | 0.44 |
| Total compounds identified |  |  | 138 | 32 |
